# Fetal Mammary Gland Development and Offspring’s Breast Cancer Risk in Adulthood

**DOI:** 10.3390/biology14020106

**Published:** 2025-01-21

**Authors:** Lawrence Mabasa, Anri Kotze, Nonhlakanipho F. Sangweni, Tarryn Willmer, Kwazikwakhe B. Gabuza, Oelfah Patel, Sylvester Ifeanyi Omoruyi, Anathi Burns, Rabia Johnson

**Affiliations:** 1 Biomedical Research and Innovation Platform, South African Medical Research Council, Tygerberg, Cape Town 7505, South Africaanathi.burns@mrc.ac.za (A.B.);; 2 Department of Medical Physiology, Stellenbosch University, Tygerberg, Cape Town 7507, South Africa; 3 Division of Cell Biology, Department of Human Biology, Faculty of Health Sciences, University of Cape Town, Cape Town 7925, South Africa; 4 School of Anatomical Sciences, Faculty of Health Sciences, University of the Witwatersrand, Parktown, Johannesburg 2193, South Africa; sylvester.omoruyi@wits.ac.za

**Keywords:** maternal environment, nutrition, breast cancer risk, mammary development, epigenetic programming, fetal programming

## Abstract

While advancements in early detection and improved access to care have significantly enhanced breast cancer survival rates, the disease remains a significant global malignancy, constituting approximately 12.5% of all new cancer cases and claiming nearly 700,000 lives in 2020. As a result, there is widespread consensus that the most sustainable solution lies in prevention. Indeed, preventive strategies, including lifestyle modifications and research into risk-reducing interventions, offer the potential to address the root causes of noncommunicable diseases such as breast cancer. While conventional wisdom has long attributed established risk factors for breast cancer to age, lifestyle, familial history, and reproductive factors, evidence highlights the maternal environment as a pivotal stage for fetal programming of disease risk, as elucidated in the developmental origins of health and disease (DOHaD) framework. Consequently, a growing body of research has been focused on elucidating epigenomic signatures that influence fetal development while shaping health outcomes and susceptibility to diseases later in life. This review aims to identify fetal mammary developmental genes that have been implicated in breast cancer etiology and the potential interplay of maternal environment in epigenetic programming of breast cancer risk in adulthood.

## Simple Summary

Breast cancer remains a significant global health challenge, accounting for approximately 12.5% of all new cancer cases and nearly 700,000 deaths in 2020. While advancements in early detection and treatment have greatly improved survival rates, prevention is widely regarded as the most effective long-term solution. Preventive strategies, including healthier lifestyles and targeted interventions, aim to address the root causes of breast cancer and other noncommunicable diseases. Traditionally, breast cancer risk has been linked to factors such as age, family history, lifestyle, and reproductive history. However, emerging evidence underscores the importance of the maternal environment during pregnancy in shaping a child’s long-term health and disease susceptibility. This concept is rooted in the developmental origins of health and disease (DOHaD) framework, which highlights how early-life exposures influence lifelong health outcomes. This review delves into the biological factors involved in fetal development that may contribute to breast cancer risk. It also explores how the maternal environment, particularly maternal nutrition, could play a pivotal role in modulating these factors to potentially reduce breast cancer susceptibility later in life.

## 1. Introduction

Breast cancer remains the predominant neoplastic condition among women, constituting approximately 12.5% of the total number of new cancer cases in the year 2020 worldwide. More concerning is the fact that within the same time frame, breast cancer resulted in approximately 700,000 fatalities, positioning it as the fifth leading cause of mortality attributed to cancer [1]. Modifiable risk factors for breast cancer include obesity, alcohol consumption, hormone therapy, and a sedentary lifestyle [2]. However, the most commonly discussed risk factors include familial history, genetic mutations, early onset of menstruation, late age of menopause, shortened breastfeeding period, late age of first full-term pregnancy, nulliparity, and low parity [3]. It has been suggested that fuller differentiation of the mammary gland during the first pregnancy, characterized by a specific genomic profile, has a protective effect against breast cancer [4]. This may be due to hormonal and genomic factors that impact the breast’s morphological and functional characteristics, especially during the heightened needs for milk production. Although current focus is largely on the regulation of postnatal mammary gland development, it is important to acknowledge that this development begins as early as the embryonic stage. In fact, accumulating evidence supports the notion that occurrences associated with fetal mammary development may be connected to the etiology of breast cancer. Indeed, studies have reported dysregulation of crucial fetal mammary developmental genes [e.g., T-box transcription factors (*TBX2* and *TBX3*)] in human breast cancer cells in vitro [5,6].

Moreover, research evidence pinpoints the maternal environment as a critical factor in fetal programming of health and diseases, a phenomenon widely recognized as the Developmental Origin of Health and Diseases (DOHaD) framework [7]. Indeed, numerous studies have underscored the critical role of maternal diet on fetal development, with evidence linking it to long-term health outcomes, including the risk of developing breast cancer. For instance, a study by Hilakivi-Clarke et al. [8] demonstrated that a maternal diet high in fat during pregnancy led to an early onset of puberty as well as an increased risk of breast cancer in female offspring. This could be attributed, in part, to epigenetic programming, with modifications such as DNA methylation playing an essential role. Although reversible, if conferred during the early developmental stages of life, DNA methylation changes may be inherited (i.e., genomic imprinting) to influence functional outcomes long after the inductive events have occurred.

DNA methylation reactions are known to depend on methyl groups generated by one-carbon metabolism, a pathway that, in turn, relies on the availability of methyl donor nutrients (MDNs) such as vitamin B_12_ and folic acid. Interestingly, researchers have previously shown that maternal diet supplemented with MDN (including vitamin B_12_, methionine, choline, and folic acid) lowers the susceptibility of offspring to developing chemically induced breast cancer [9,10]. These findings suggest that maternal MDN reduces the offspring’s susceptibility to developing mammary tumors, partly through epigenetic modification of genes involved in cellular growth.

While advancements have been made in understanding the maternal environment’s role in shaping offspring health, significant gaps remain in the study of fetal mammary gland development and its impact on breast cancer risk. Existing research is limited by a scarcity of data on the molecular pathways governing embryonic mammary development and how maternal factors, such as diet and epigenetic modifiers, influence these processes. Moreover, most studies focus on postnatal or pubertal mammary gland development, overlooking critical prenatal stages where early genomic and epigenomic signatures may predispose individuals to cancer. Questions such as how specific genes, like *TBX3* or components of the WNT and FGF signaling pathways, are epigenetically regulated by maternal nutrition, and their implications for lifelong breast cancer susceptibility, remain underexplored. This review aims to synthesize current knowledge, identify gaps, and propose potential mechanisms linking fetal mammary gland development to adult breast cancer risk, offering insights for future research and prevention strategies. We aim to identify fetal mammary developmental genes implicated in breast tumorigenesis from the literature and propose a mechanism by which the maternal environment influences the fetal programming of breast cancer risk through epigenetic regulation of gene transcription.

A comprehensive literature survey was conducted using platforms such as PubMed, EMBASE, MEDLINE, Google Scholar, and Web of Science. To ensure an extensive search, the following terms and their alternatives were utilized with Boolean operators: (“mammary glands” OR “breast”) AND (“fetal growth” OR “fetal development”) AND (“mammogenesis” OR “breast development”). Additional searches included “epigenetics” OR “DNA methylation”, as well as “fetal programming” OR “developmental origins of health and disease” OR “DOHaD.” Keywords related to maternal and offspring health, such as “pregnancy” OR “prenatal” OR “gestation” OR “maternal” OR “women” OR “mother”, were combined with “offspring” OR “child” OR “children” to capture relevant studies.

The search process was conducted in two steps. In both steps, Robinson [11] served as a key reference to identify genes implicated in fetal mammary development. Genes such as T-box transcription factors, *TBX2* and *TBX3*, as well as those involved in the *WNT*/β-catenin and fibroblast growth factor (FGF) signaling pathways, were included in the first search to identify studies highlighting their roles in fetal mammary biology. Approximately 16,580 articles were initially retrieved. After filtering to only include animal studies (due to the limited availability of fetal mammary development models in humans), the dataset was reduced to 10,016 articles. Further refinement to include only research and systematic review articles narrowed the results to 1403, comprising 556 primary research articles. Subsequently, an additional filter was applied to include only studies focused on females, reducing the total to 135 articles for abstract screening and full-text review.

For the second search, the same protocol was employed, with the inclusion of “breast cancer” OR “breast tumors” OR “mammary carcinogenesis” OR “breast neoplasm” as additional search terms without the fetal mammary development terms. This was meant to identify studies implicating the aforementioned genes in breast tumorigenesis, as well as the potential role thereof of “epigenetics” OR “Epigenomics” OR “DNA methylation”.

## 2. Developmental Origin of Health and Diseases

The DOHaD concept best represents the essence of developmental plasticity. This phenomenon describes a critical developmental window (in utero) during which genomic signatures are highly sensitive to environmental stimuli, leading to morphological and physiological changes that can influence disease susceptibility and pathological outcomes in adulthood [11]. The Dutch famine has been the best illustration of this phenomenon, highlighting the impact of adverse environmental insults during pregnancy on birth weight and the long-term health of the offspring. Indeed, the sudden onset and relief from famine in a previously well-nourished population offered an opportunity to study pregnancy-related malnutrition without the confounding factors of pre- and postnatal environmental factors [12]. Pertinently, pregnancy during this period was associated with low birth weights, and these offspring were subsequently shown to have a heightened risk of developing cardiometabolic disorders, in part due to epigenetic modifications (in particular, DNA methylation) of genes involved in growth and metabolism [7,13,14]. In other studies, women who were conceived during the Great Leap Forward famine were reported to have an increased incidence of breast cancer as compared to women who were conceived before or after the famine [15]. Additionally, Silver and colleagues employed genome-wide DNA methylation profiling to examine a community in rural Gambia [16]. This setting, marked by seasonal changes in food availability and metabolic needs, acts as a natural opportunity to explore how peri-conceptional conditions, including maternal nutrition, affect the epigenetic development of offspring. In this context, the loss of imprinting on VTRNA2-1, a tumor suppressor gene, was proposed as a possible link between early embryonic nutrition, epigenetic changes (such as DNA methylation), and the development of diseases later in life. Most recently, a Japanese birth cohort study has been initiated to assess whether the changes in environmental factors due to the COVID-19 pandemic influence fetal programming of health in the children. Such an endeavor has the potential to add to the body of knowledge concerning the role of adverse natural disasters and associated maternal environmental changes on health trajectory long after the inductive events have occurred [17].

The role of maternal nutrition in fetal programming of health and disease risk was further illustrated in a recent study by Mikołajczyk-Stecyna and colleagues, albeit conducted using an animal model [18]. The researchers demonstrated that maternal choline supplementation in dams with fatty liver disease reduced the risk of their offspring developing the condition compared to offspring of dams that received basal levels of choline. Choline, a crucial methyl donor in one-carbon metabolism, generates methyl groups used in DNA methylation, a process that regulates gene transcription. It is essential for fetal development and serves as a precursor for biomolecules such as phosphatidylcholine, a key component of cell membranes.

In the context of breast cancer, animal studies have demonstrated that maternal supplementation with methyl donor micronutrients (vitamin B_12_, folic acid, methionine plus choline) can reduce the susceptibility of rat offspring to developing breast cancer in adulthood [9,19,20]. However, the underlying mechanisms driving this protective effect remain largely unexplored. Moreover, while several fetal mammary developmental genes have been implicated in breast tumorigenesis, there is limited information on how the maternal environment influences their expression patterns and, consequently, the offspring’s risk of breast cancer later in life.

## 3. Mammary Gland Development

The mammary gland is an intricate organ that develops from the apocrine sweat glands, encompassing various cell types such as epithelial cells, adipocytes, vascular endothelial cells, fibroblasts, and immune cells. The epithelial cells can be categorized into basal cells (including myoepithelial cells) and luminal cells (encompassing ducts and the lumen). The development of the mammary gland takes place through two distinct phases, ductal and secretory, and these are further divided into the following stages: pubertal, pregnancy, lactation, and involution [21]. While developmental processes during the embryonic stage produce the rudimentary ductal structure of the gland that is present postnatally under the control of sex hormones such as estrogen and growth hormones [e.g., insulin-like growth factor 1 (*IGF-1*)], pubertal mammary development yields a ductal tree that fills the fat pad. During pregnancy, in preparation for milk production, the alveoli are formed under the stimulatory influence of progesterone and prolactin. As the demand for milk recedes, involution is initiated at weaning in order to remodel the glands to promote the next cycle [21,22]. In this review, emphasis will be placed on embryonic mammary gland development due to its potential significance in influencing the risk of breast cancer in adulthood through fetal programming.

### 3.1. Embryonic Mammary Gland Development

#### 3.1.1. Structural Characteristics

**Figure 1:**
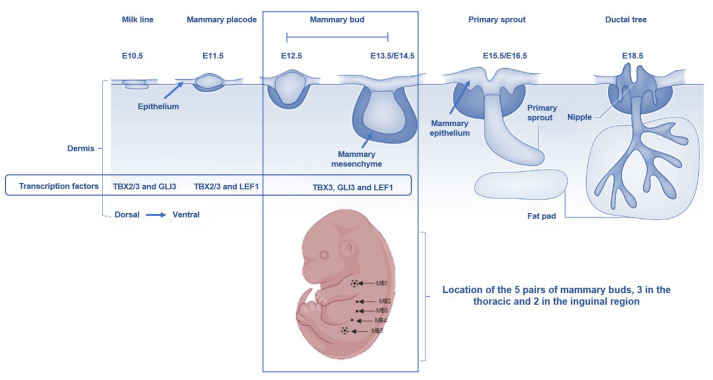
Stages of murine fetal mammary gland development, including the formation of milk line by embryonic day 10.5 (E10.5), mammary placodes by E11.5, mammary buds (5 pairs from which nipples are formed) between E12.5 and E13.5/E14.5, primary sprout by E15.5/E16.5 and ductal tree by E18.5 (adapted from Robinson [23]).

For the purpose of this review, mice embryonic mammary gland development will be discussed as available evidence indicates that there are similarities with human embryos [23]. The gestation period of mice spans from 19 to 21 days (3 weeks), during which embryonic mammary gland development undergoes distinct stages. Murine mammary gland development commences at embryonic day 10.5 (E10.5), marked by the formation of milk lines that extend from the forelimb to the hindlimb (Figure 1). By E11.5, these milk lines transform into five pairs of placodes (the future sites of nipples), coinciding with the condensation of mammary mesenchyme. Subsequently, the mammary placodes descend into the dermis, and the mammary mesenchyme organizes into concentric layers around the mammary bud (MB1–5)-like structures in 3 thoracic and 2 inguinal regions [24,25]. By E15.5, the mammary epithelium begins to proliferate, with the primary sprout pushing through the mesenchyme towards the fat pad. At E18.5, the ductal system is established, with the cells of the mesenchyme forming the nipple, while the ductal tree is fully embedded into the fat pad. This sequential process of development is essential for establishing the structural and functional framework of the mammary gland, ensuring its ability to support lactation and neonatal nutrition postnatally.

#### 3.1.2. Gene Regulatory Networks

Embryonic mammary gland development is a tightly regulated process that progresses through distinct stages, guided by various signaling pathways, transcription factors, and growth factors. In the initial phase of mammary development, the Wnt/β-catenin signaling pathway facilitates the proliferation of epithelial cells and the development of mammary placodes [26]. Next, fibroblast growth factor (FGF) and bone morphogenetic protein (BMP) signaling pathways play a vital role in the creation of the ductal system [23]. As development continues, crucial transcription factors, including *TBX3*, guide the fate and differentiation of epithelial cells, promoting the transition from simple epithelial structures to more intricate branching ducts [27]. Additionally, the role of estrogen signaling is significant during this stage [28]. The Notch signaling pathway also plays a crucial role in balancing cell differentiation and proliferation, thereby ensuring effective ductal morphogenesis [29].

As indicated above, the development of various features of the mammary glands is a highly regulated process involving proteins such as T-box transcription factors, *TBX2* and *TBX3*, and genomic signals including fibroblast growth factors (*FGFs*) and *WNT* signaling (Table 1). *TBX3* is primarily expressed in the mesenchyme and epithelial buds of the mammary glands, with initial detection in the nascent mammary buds’ epithelium at E11.5 [30,31]. Heterozygous mutation of *TBX3* in mouse embryos previously resulted in reduced placode formation and branching morphogenesis in the first three pairs of mammary glands without affecting the fourth and fifth pairs [30,31].

**Table 1:** Selected genes involved in mouse embryonic mammary development.

Species	Key Mammary Developmental Gene	Role	Source
Mice	*TBX3*	Stimulates mammary placodes induction and branching morphogenesis	[32]
Mice	*WNT-10b*	Essential for initiation of mammary morphogenesis as well as promotion of placode formation	[26]
Mice	*FGF10*	Induction of mammary placodes 1, 2, 3, and 5	[33]
Mice	*LEF1*	Plays a role in the formation of mammary placodes	[26,33,34,35]
Mice	*BMP4*	Essential for the outgrowth of mammary buds	[36]
Mice	*PTHrP*	Stimulates ductal development	[36]
Mice	*Nrg3*	Confers mammary phenotype specification to mesenchymal and epithelial progenitor cells	[37]
Mice	*GLI* *3*	Required for the formation of mammary placode 3 and 5	[38]

The *WNT* signaling pathway is widely regarded as a critical component of mammary gland development, as evidenced by accelerated placode development amid elevated expression of placode markers, such as *WNT-10b* [26]. Indeed, transgenic embryos expressing Dickkopf 1 (*DKK1*, a *WNT* inhibitor) showed a lack of placode formation, further emphasizing the significance of *WNT* signaling in embryonic mammary gland development.

The FGF family, consisting of at least 22 proteins, including *FGF10*, plays a pivotal role in mammary placode induction [24]. For instance, *FGF10−/−* embryos in a C57BL6 mice background exhibited the absence of mammary placodes 1, 2, 3, and 5, while placode 4 was present [24]. As a result, it was postulated that a different ligand for *FGF10*’s main receptor (FGFR2b) could be responsible for the induction and maintenance of placode 4.

Lymphoid enhancer-binding factor 1 (*LEF1*) is a transcriptional mediator of the canonical *WNT* signaling pathway known to induce the early development of the mammary glands during the embryonic stage [39]. Indeed, deletion of *LEF1* has previously been shown to precede the disappearance of mammary placodes 1, 4, and 5, and subsequently, the lack of mammary bud formation [24,26,34,35].

Further, an interaction between bone morphogenetic protein 4 (*BMP4*) and parathyroid hormone-related protein (*PTHrP*) has also been identified as a key regulator of *WNT* signaling-directed stimulation of embryonic mammary bud formation. Indeed, a study by Hens and colleagues [36] previously reported that *PTHrP* sensitizes mesenchymal cells to the action of *BMP4* by upregulating the expression of the BMP1A receptor, which then suppresses hair follicle formation near the developing mammary buds and nipple while also promoting mammary bud sprouting [36]. In support, mutations in *PTHrP* were previously shown to impair normal development of the mammary glands in humans and rodents [36,40]. Interestingly, while previous reports indicated that overexpression of *BMP4* in mice embryos induces *LEF1* expression, in contrast, that of *TBX3* was reduced; overexpression of *TBX3* was also shown to have a similar effect on *BMP4*, with the latter leading to an expansion of the mammary epithelium [41].

The expression of Neuregulin3 (*NRG3*) and its receptor, *ERBB4*, has also been implicated in mammary bud induction, as they have been detected within the presumptive mammary gland region by E11 prior to the morphological appearance of mammary buds [42]. In agreement, researchers have demonstrated the absence of epithelial and mesenchymal progenitor cell aggregation and specification crucial for early mammary gland induction in *NRG3* mice mutant embryos [37].

Lastly, *GLI3*, a zinc finger transcription factor, is a known mediator of the Hedgehog (Hh) signaling pathway, playing a pivotal role in processes such as embryogenesis, tissue homeostasis, mammary development, and tumorigenesis. Indeed, removal of *GLI3* function in mice resulted in aberrant placode formation and lack of two pairs of mammary buds [43].

In conclusion, a deep comprehension of the intricate molecular mechanisms regulating embryonic mammary gland development presents a significant opportunity to delve into the exploration of fetal programming regarding breast cancer risk in adulthood. The evolving paradigm of developmental origins of health and disease (DOHaD) logically directs attention towards investigating the potential influence of the maternal environment on epigenetic modifications of genes involved in fetal mammary development and their subsequent implications for breast cancer susceptibility in later life. It is logical to assume that dysregulation or mutations in key mammary gland development regulators such as *TBX3*, FGFs, WNT signaling components, *LEF1*, *BMP4*, *PTHrP*, *NRG3*, ERBB4, and *GLI3* during critical stages of mammary gland development may imprint lasting effects on the mammary gland structure and function, potentially predisposing individuals to heightened susceptibility to breast cancer later in life. This perspective underscores the importance of unraveling the complex interplay between early-life environmental exposures and long-term health outcomes, offering avenues for preventive measures to mitigate breast cancer risk across the lifespan.

#### 3.1.3. Comparison to Human

However, it should be noted that mouse and human mammary glands exhibit significant architectural, hormonal, and stromal differences. For example, while mouse mammary glands feature a simpler ductal structure with terminal end buds rich in stem cells, human mammary glands are more complex, composed of lobules branching from larger ducts [44,45]. Mouse mammary glands are primarily involved in lactation during the reproductive phase, with relatively low levels of branching and alveolar development outside of pregnancy and lactation [46]. Human mammary glands, on the other hand, exhibit extensive baseline branching within the terminal ductal lobular units (TDLUs), and post-lactation, TDLUs remain extensively branched. Additionally, while some pathways, such as Wnt and Notch signaling, are conserved across species, their regulation and interactions with other pathways may differ. Disparities in hormonal milieu and stromal composition also impact mammary gland biology, potentially modulating stem cell behavior and disease susceptibility. Indeed, human breast stroma contains abundant fibroblasts with distinct morphogenetic roles, whereas mouse mammary fat pads are predominantly composed of adipose tissues. This complexity poses challenges in understanding functional units and disease mechanisms. Recognizing these interspecies variations is crucial for accurate disease modeling and the development of targeted therapies tailored to human mammary gland biology while leveraging insights from mouse models.

### 3.2. Fetal Mammary Developmental Genes in Breast Cancer Risk

#### 3.2.1. Classification of Breast Cancer

Breast cancer is a genetically and clinically heterogeneous disease comprising many subtypes. Whilst the classification of these subtypes has evolved over the past decade, they are most commonly classified based on the expression status of the three hormone receptors, namely estrogen receptor (ER), progesterone receptor (PR), and human epidermal growth factor (HER2) [47]. Accordingly, breast cancer is categorized into four widely recognized subtypes. The luminal A subtype is estrogen- (ER) and/or progesterone (PR) receptor-positive, accounting for the majority of all breast cancer cases with the best prognosis [48]. Luminal B is ER and/or PR positive, may exhibit positive or negative expression of human epidermal growth factor receptor 2 (HER2), and accounts for approximately 20% of breast cancer cases. These tumors are of higher grade and worse prognosis compared to Luminal A tumors [49]. Luminal A and Luminal B breast cancer subtypes further differ in their Ki-67 expression, with Luminal A showing low Ki-67 levels (<20%), while Luminal B exhibits high Ki-67 levels (>20%). HER2-positive tumors, which grow faster than the luminal subtypes, account for approximately 10–15% of breast cancer cases and are characterized by high HER2 expression with absence of ER and PR [50]. Triple-negative breast cancers (TNBCs) are the most aggressive in nature and lack the expression of all three receptors. They represent around 14% of breast cancer incidence and are most common among women under 40 years of age as well as women of African descent [51].

These four breast cancer subtypes exhibit distinct biological and clinical behavior and, not surprisingly, require distinct and tailored therapeutic interventions [52]. ER-positive breast cancers require first-line adjuvant therapies such as tamoxifen and aromatase inhibitors, which target and block the ER [48]. HER2-positive breast cancers are normally treated using HER2-targeting monoclonal antibodies such as trastuzumab or combination therapies [53]. TNBCs represent the most proliferative subtype, and since they lack well-defined clinical targets, their treatment options are limited to standard chemotherapy regimens such as taxanes and anthracyclines, thus providing evidence that novel prognostic biomarkers and therapeutic targets are required for the treatment of individual breast cancer subtypes [54].

Invasive ductal carcinoma (IDC) is the most commonly diagnosed histologic subtype, accounting for over 70% of all invasive breast cancers, followed by invasive lobular carcinoma, which represents around 15% of all invasive breast cancers [55,56]. In a study of about 171,881 breast cancer patients, 84% had IDC; of these, 82% were ER-positive, 72% were PR-positive, and 84% were HER2-negative, confirming that IDC is the most common invasive breast cancer, with Luminal A accounting for the majority of the tumors [55]. Interestingly, previous studies have also demonstrated that individuals diagnosed with ER- and PR-positive ILC and IDC display comparable quantitative profiles of *ER* and *PR* expression [57]. This suggests that, to an extent, the expression of ER/PR is improbable to act as a confounding factor in research focused on the chemo-sensitivity between ILC and IDC.

#### 3.2.2. Role of Fetal Developmental Genes in Breast Tumorigenesis

For this section, data collected from mostly human subjects will be discussed. Of interest is a study by Ciriello et al. [56] that profiled genomic alterations that discriminate between IDC and ILC in order to provide insight into different breast cancer subtypes and potential therapeutic targets. In this study, *TBX3* mutation (Table 2), loss of e-cadherin (*CDH1*), and activated protein kinase B, also known as *AKT*, were identified as characteristic features strongly associated with ILC, while mutation of *GATA3* was predominant in luminal A IDC [56]. While the loss of CDH1 has been shown to promote tumor invasion and metastasis, especially in ER-positive breast cancer, AKT is known to stimulate tumor cell growth and proliferation by possessing anti-apoptotic properties [56,58,59,60]. Interestingly, *TBX3* has been shown to promote cancer cell migration via activation of inhibitor of DNA binding 1 (ID1), a negative regulator of CDH1 [61].

**Table 2:** Embryonic mammary developmental genes implicated in breast cancer etiology.

Population/Tissue	Gene	Breast Cancer Subtype	Role in Cancer Biology	Source
Human	*TBX3*	Mutations associated with ILC	Promotes cancer cell migration and invasion	[56,62,63]
Human	*GLI* *3*	Highly expressed in invasive carcinomas	It is a protein involved in the Hh signaling pathway and has been shown to promote proliferation, migration, and invasion	[64]
Human	*WNT-10b*	Downregulated in some human IDC	Important in the oncogenic transformation of breast epithelial cells	[65]
Human	*WNT-6*	Upregulated in human IDC	Important in the oncogenic transformation of breast epithelial cells	[65,66]
Human	*LEF1*	Upregulated in human breast tumors, especially in Luminal A cancer	*LEF1* is a downstream component of the WNT signaling with pro-invasive activity in breast cancer	[67,68]
Human and in vitro	*FGF10*	Elevated in mice invasive subcutaneous mammary tumors, primary human breast carcinomas, MCF-7 and MDA-MB-231 human breast cancer cells	Postulated to be a proto-oncogene and antiapoptotic protein	[69,70]
Human and in vitro	*BMP4*	Upregulated in TNBC (e.g., MDA-MB-231 cells) and human breast tumors	Promotes cell proliferation in TNBC while in contrast, it induces cell cycle arrest in IDC. Indeed, in luminal breast cancer, *BMP4* loss was associated with tumor aggression	[71,72]
Human	*PTHrP*	Overexpressed in TNBC and IDC	Promotes proliferation and metastasis in triple-negative breast cancer and IDC	[73,74]
Human	*NRG3*	Expressed in some low-grade IDC	ErbB/HER receptor ligand that binds to HER4 and is known to possess anti-proliferative properties on some breast cancer types	[75,76]

Further studies have shown that *TBX3*, in turn, is activated by AKT3 via phosphorylation on the serine residue 720 [77]. On the other hand, GATA3 has been shown to possess oncogenic properties in IDC breast cancer [78]. In this regard, using cross-linking chromatin immunoprecipitation, *TBX3* has also been shown to regulate the expression of *GATA3* as well as that of *GLI3* by binding to putative DNA enhancer sequence sites [79]. *GLI3* in particular is required for the formation of mammary placodes in utero, and its knockdown in invasive MDA-MB-231 human breast cancer cells has previously been shown to inhibit tumor proliferation, migration, and invasion [64].

The *WNT*/β-catenin signaling is a highly conserved pathway comprising growth stimulatory factors. Its dysregulation has been implicated in the pathogenesis of various diseases, including breast cancer. Briefly, in the absence of an external signal [i.e., *WNT* protein binding to the low-density lipoprotein receptor protein 5 or 6 (LRP) and frizzled (FZD) receptors], β-catenin is phosphorylated by a destruction complex [comprising tumor suppressors, Axin and adenomatous polyposis coli (APC), the serine/Thr kinase GSK-3 and CK1 (both of which are responsible for β-catenin phosphorylation)], which then leads to it being ubiquitinated and degraded by proteosomes. The degradation of β-catenin prevents it from accumulating in the cytosol and the nucleus, leading to transcriptional silencing as Groucho, a transcriptional repressor, remains bound to transcription regulators, TCF/*LEF1*. However, the presence of *WNT* protein and its binding to LRP and coreceptor FZD triggers a cascade of events, which leads to unphosphorylated β-catenin displacing Groucho from TCF/*LEF1*, which then results in transcription of *WNT* target genes. Indeed, overexpression of *LEF1* has previously been shown to enhance breast tumor invasion, although the study did not assess the related activity of downstream proteins [68]. Further, studies have shown that several proteins that can successfully disrupt the binding of TCF to β-catenin may prevent cancer cell proliferation and migration by suppressing the transcription of genes such as *C-MYC* and cyclin D1 [80]. While C-MYC is involved in facilitating a pro-tumor microenvironment, cyclin D1 is essential in cell cycle progression, DNA synthesis, and cell proliferation. Indeed, a study by Yang et al. [81] reported that suppression of *WNT*5B led to G0/G1 cell cycle arrest and subsequently, a reduction in TNBC cell growth. Further, a study by Ain et al. [65] revealed an overexpression of *WNT*-6 and, in contrast, a downregulation of *WNT-10b* in human breast cancer tissues as compared to adjacent normal breast tissues. Both proteins were reported to be important in the oncogenic transformation of breast epithelial cells [65,66].

The canonical fibroblast growth factor receptor (FGFR) pathway is a signaling cascade that involves multiple downstream kinases, such as the PI3K/AKT pathway. These kinases, when activated, have been shown to influence functions such as cell proliferation, survival, growth, differentiation, and migration [82]. Importantly, FGFR signaling is known to play a key role in angiogenesis and early embryogenic developmental processes, while deregulation of the pathway has also been implicated in the pathogenesis and progression of various diseases. Indeed, *FGF10*, a member of the canonical FGF7 subfamily of proteins, is elevated in highly vascularized invasive subcutaneous mice mammary tumors and several human breast cancer subtypes (both Luminal A and TNBC) [69,70].

The bone morphogenetic proteins (BMPs) are multifunctional cytokines that belong to the transforming growth factor superfamily and confer their signaling via receptors of serine–threonine kinase types I and II, along with their intracellular downstream effectors, such as SMAD proteins [83]. As in FGF, these proteins are known to play a role in cellular functions such as differentiation, proliferation, survival, and migration, but have also been implicated in the pathogenesis of cancer [84]. Indeed, *BMP4* was previously demonstrated to promote cell proliferation in TNBC MDA-MB-231 cells, while in contrast, it was reported to induce cell cycle arrest in MCF-7 human breast cancer cells [71]. Further, the study showed that *BMP4* promotes colony formation and migration in MDA-MB-231, but it did not affect MCF-7 cells. Interestingly, a study by Eckhardt and colleagues showed that loss of *BMP4* or its downstream effector, SMAD7, promotes breast cancer metastasis [72]. These contradictory findings suggest that the effect of *BMP4* on cancer growth and migration may be dependent on the tumor subtype and requires further assessments. Interestingly, activation of both the WNT/β-catenin and FGF signaling pathways have been shown to directly upregulates the expression of *TBX3*, thereby leading to breast cancer stem cell expansion. Additionally, *BMP4* overexpression has been shown to inversely correlate with *TBX3* expression in mammary tissues; however, it remains unclear whether this relationship holds true in breast cancer, particularly given that both *BMP4* and *TBX3* exhibit pro-proliferative properties in breast cancer [41,85].

Parathyroid hormone-related protein (*PTHrP*), *NRG3*, and *GLI3* are additionally recognized as having a role in breast cancer pathogenesis and prognosis. Indeed, overexpression of *PTHrP* was positively associated with poor survival in a cohort with newly diagnosed TNBC [73]. It has further been reported that *PTHrP* affects breast cancer growth by binding to and suppressing the expression of leukemia inhibitory factor receptor (LIFR), a cytokine receptor known to inhibit cancer cell differentiation via activation of signal transducer and activator of transcription (STAT3) of the Janus kinase signal transducer and activator of transcription (JAK-STAT) [74]. The *NRG3* protein is an ERBB/HER receptor ligand known to inhibit IDC proliferation and invasiveness by binding and activating HER4, a receptor tyrosine kinase required for the evolution of mammary glands [75,76].

Taken together, the interplay of molecular pathways in breast cancer pathogenesis underscores the complexity of this disease. Studies such as that by Ciriello et al. shed light on the nuanced genomic alterations distinguishing between different breast cancer subtypes, offering potential therapeutic avenues [56]. Particularly noteworthy are findings implicating *TBX3* in various facets of tumorigenesis, including invasion, proliferation, and metastasis. Furthermore, the dysregulation of signaling cascades like WNT/β-catenin and FGFR highlights the multifaceted nature of breast cancer biology and the need for tailored prevention and treatment approaches. These insights underscore the need for further research to elucidate subtype-specific vulnerabilities and develop targeted interventions, ultimately advancing prevention and patient outcomes.

Additionally, the reported role in breast cancer of fetal mammary developmental genes, such as *TBX3*, *WNT-10b*, *FGF10*, and *LEF1*, highlights their potential clinical relevance as biomarkers and therapeutic targets. Dysregulation of these genes, often through epigenetic modifications, could serve as early diagnostic and prognostic indicators, particularly for aggressive subtypes like triple-negative breast cancer. Also, insights into how maternal factors, including diet, influence the epigenetic programming of these genes also present opportunities for preventive strategies. Nonetheless, further research is needed to assess the implication of this epigenetic phenomenon on the susceptibility of offspring to developing breast cancer, as well as the responsiveness of the tumor to therapy.

## 4. Molecular Basis for Fetal Programming of Breast Cancer Susceptibility

Studying fetal programming of disease risk entails investigating how early developmental events influence the predisposition to diseases later in life. For example, maternal nutrition has been shown to influence DNA methylation-mediated gene transcription regulation, resulting in lasting effects into adulthood that significantly impact later-life health outcomes such as metabolic syndrome and breast cancer risk [86,87,88]. From the above section, evidence revealed intriguing roles of fetal mammary developmental genes, such as the T-box transcription factor, *TBX3*, in breast tumorigenesis. These genes play critical roles in orchestrating various aspects of embryonic mammary gland development, including cell proliferation, differentiation, and tissue organization.

However, our understanding of the complex molecular regulatory network governing fetal mammary gland development remains incomplete, leaving a gap in our understanding of fetal programming of breast cancer risk. Enhanced comprehension of these molecular pathways is crucial for unraveling the complex interplay between prenatal factors, mammary gland development, and the long-term susceptibility to breast cancer. Thus, further investigation into this intricate network is imperative to advance our understanding and potentially inform preventive strategies and therapeutic interventions for breast cancer. In the following section, we present a hypothetical research avenue to explore how maternal environmental cues, particularly maternal nutrition, influence fetal mammary biological processes, potentially shaping breast cancer susceptibility in adulthood.

### 4.1. Fetal Mammary Stem Cells

Experimental evidence supports the hypothesis that some breast cancers originate from a fetal environment that induces persistent epigenetic modification and the expression of genes that regulate cell proliferation, apoptosis, survival, and differentiation [87]. In this context, the contribution of stem cells to fetal programming of health and disease risk cannot be overlooked. Stem cells, with their capacity for self-renewal and differentiation, are central players in fetal programming of disease risk, responding to environmental cues during development. Although rare and seldom divided in adult mammary glands, mammary stem cells possess significant proliferative capacity and self-renewal upon cell division [87]. In fact, researchers have documented the ability of a single mammary stem cell to generate a significant number of specialized cells, effectively recreating the entire functional gland [89,90]. Mutations in the mammary epithelial stem cell population hold greater relevance as they are permanently preserved and inherited by all subsequent progeny, potentially posing a significant risk for tumorigenesis due to their extensive proliferative capacity and self-renewal potential [91].

Moreover, the advent of DNA methylation has stimulated an exciting research avenue in fetal programming of health and disease risk. Due to its potential to maintain cellular memory during self-renewal, DNA methylation has been shown to play a role in mammary and cancer stem cells’ maintenance [92]. Interestingly, *TBX3* has been shown to maintain mouse embryonic stem cell pluripotency in the same manner that it stimulates breast cancer stem cells [93,94]. One of the molecular mechanisms through which *TBX3* confers its breast tumor-promoting properties involves its interaction with HDAC 1, 2, 3, and 5, the consequence of which is to downregulate key cell cycle regulators such as tumor suppressor p14^ARF^ [93]. Despite this, there remains a notable gap in understanding the epigenetic modulation of *TBX3* and other factors involved in mammary stem cell maintenance, particularly their role in breast cancer susceptibility and progression.

Nonetheless, studies in other cancer types provide valuable insights. In bladder cancer, methylation of *TBX2* and *TBX3* has been associated with disease progression [94]. In colorectal cancer, *GLI3* methylation has been identified as a potential blood-based biomarker for detection [95], while aberrant methylation of *WNT-6* has also been documented [87]. Additionally, promoter methylation of *NRG3* has also been observed in some colorectal cancer patients [96].

In the context of breast cancer, limited but noteworthy findings have emerged. Indeed, methylation of another member of T-Box transcription factors, TBX15, was detected in triple-negative breast cancer as compared to non-triple-negative breast cancer, suggesting that it might be involved in the non-responsiveness to hormonal therapy [97]. While TBX15 is linked to belly coat color in murine, its expression and that of *TBX3* have been shown to be inversely regulated by *BMP4* expression during the early mammary developmental phase [41]. Further, methylation of *LEF1* has been linked to a lack of response to neoadjuvant chemotherapy in triple-negative breast cancer patients, highlighting its potential as a predictive biomarker for treatment resistance [98]. Moreover, promoter methylation of *PTHrP* was positively correlated with its expression in vitro, and this was proposed as a potential biomarker for breast cancer progression [99].

Nonetheless, future research should address the significant gap in understanding the methylation of these genes in breast cancer. Expanding studies to include methylation profiles in the context of fetal programming of breast cancer susceptibility and progression could lead to the identification of novel preventive, diagnostic, and prognostic biomarkers and the development of targeted epigenetic therapies, particularly for challenging subtypes such as triple-negative breast cancer.

### 4.2. Potential Avenue

**Figure 2:**
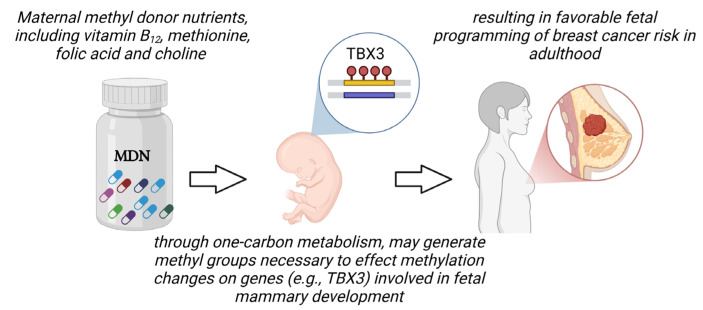
Exposure to maternal methyl donor nutrients could potentially influence breast cancer risk in offspring, in part, via epigenetic regulation of *TBX3* gene transcription.

The dietary choices made during pregnancy profoundly influence fetal development, as optimal nutrition is vital for the growth and well-being of the unborn child. A balanced diet rich in essential nutrients is paramount in ensuring the baby’s future health trajectory. It is understood that maternal environment, comprising diet, lifestyle choices, and chronic diseases, plays a pivotal role in orchestrating the epigenetic control of developmental genes and their involvement in shaping the long-term health of the offspring (Figure 2). Of particular importance is the inclusion of folic acid, crucial for the development of the baby’s brain and spine and playing a pivotal role in preventing neural tube defects (NTDs). Methyl donor nutrients are essential dietary components that contribute methyl groups for various biochemical processes crucial for gene regulation, protein synthesis, and metabolic functions. These nutrients, including folate, choline, methionine, and vitamins B_6_ and B_12_, play fundamental roles in the one-carbon metabolism pathway, where methyl groups are transferred to DNA, proteins, lipids, and neurotransmitters. Through this process, methyl donor nutrients influence epigenetic modifications, such as DNA methylation, which regulate gene expression patterns without altering the underlying genetic code. Consequently, methyl donor nutrients are integral for maintaining cellular integrity, supporting optimal development, and modulating physiological responses, with implications for overall health and disease susceptibility.

Interestingly, researchers have previously shown that maternal diet supplemented with methyl donor nutrients, including vitamin B_12_, methionine, choline, and folic acid, lower the susceptibility of offspring to developing chemically induced breast cancer [9,10]. While this is interesting, these studies lacked mechanistic exploration. It is plausible to postulate, as illustrated in Figure 2, that maternal methyl donor nutrients, through one-carbon metabolism, may impact DNA methylation reactions during the embryonic stage. In particular, it is hypothesized that maternal methyl donor nutrients may influence DNA methylation regulation of *TBX3* in embryonic mammary stem cells; these cells possess the ability to self-renew and differentiate into various specialized cell types that form the mammary gland, including luminal epithelial cells and myoepithelial cells. Once established, these epigenetic modifications are capable of being sustained through adulthood via epigenetic imprinting mechanisms. This enduring nature subsequently holds the potential to modulate *TBX3*’s control of cell cycle regulators such as p14/p19^ARF^, and subsequently, its engagement in pivotal biological processes associated with tumorigenesis, encompassing but not confined to cancer stem cell expansion, bypassing of cellular senescence, anti-apoptosis, and uncontrolled cell proliferation.

### 4.3. Mechanistic Exploration

Taking a comprehensive approach, such as whole genome bisulfite sequencing, enables researchers to delve deeply into the intricate regulatory networks governing genes associated with fetal mammary gland development and its contribution to breast cancer susceptibility in adulthood. This high-resolution epigenomic method offers a unique opportunity to unravel the dynamic interactions between genes and environmental cues during critical developmental stages. By scrutinizing gene expression patterns, epigenetic modifications, and gene-environment interactions, such as those influenced by maternal nutrition, we can pinpoint crucial windows of susceptibility and devise strategies for early intervention and prevention.

Conducting epigenome-wide assessments of fetal mammary glands at various stages of pregnancy in murine models allows for the identification of differentially methylated genes that are pivotal for mammary development. These epigenetic changes serve as sensitive indicators of how maternal environmental factors shape gene expression patterns, potentially influencing the long-term susceptibility to breast cancer in offspring. Within this framework, it becomes possible to evaluate the persistence of these epigenetic modifications and their roles in breast cancer susceptibility. Such insights are essential in understanding the interplay between environmental exposures, epigenetic regulation, and disease susceptibility, paving the way for targeted interventions and preventive measures.

### 4.4. Possible Models to Use

Given the challenges of directly studying fetal mammary development in humans, data from murine studies can serve as a valuable resource. These findings can inform the identification of candidate genes that may be relevant across different human breast cancer subtypes. This cross-species translational approach not only enhances our understanding of the developmental origins of health and disease but also offers potential targets for intervention strategies aimed at mitigating breast cancer risk.

Alternatively, a recent study developed a long-term adult stem cell-derived mammary mini gland culture system that accurately mimics the architecture, size, function, and transcriptional heterogeneity of the normal mammary gland under homeostatic conditions [100]. This system demonstrates hormone responsiveness and recapitulates key developmental stages, including puberty, the estrus cycle, lactation, and involution, while also maintaining mammary stem cells and enabling oncogene-induced tumor initiation in vitro, thereby offering a valuable experimental platform for studying complex biological processes. This approach may be useful as it is non-invasive with minimal ethical implications.

## 5. Conclusions

Breast cancer ranks among the most prevalent neoplastic conditions globally, with modifiable risk factors such as obesity, alcohol consumption, and hormone therapy contributing to its incidence. However, the focus on modifiable risk factors often overshadows the critical role of early developmental events, including embryonic mammary gland development, in shaping breast cancer susceptibility. The reviewed literature underscores the complex interplay between fetal mammary gland development, genetic regulation, and breast cancer etiology.

The concept of developmental origins of health and diseases (DOHaD) highlights the sensitivity of genomic signatures to environmental stimuli during critical developmental windows, leading to long-term physiological and pathological outcomes. Studies examining the impact of maternal nutrition, specifically methyl donor nutrients (MDN) supplementation, on offspring susceptibility to breast cancer underscore the potential role of epigenetic programming in disease risk.

Furthermore, investigations into the genetic regulatory networks underlying embryonic mammary gland development reveal key genes and signaling pathways implicated in breast cancer pathogenesis. T-box transcription factors (*TBX2* and *TBX3*), WNT signaling, fibroblast growth factors (FGFs), bone morphogenetic proteins (BMPs), and parathyroid hormone-related protein (*PTHrP*) are among the molecular players identified in both mammary gland development and breast cancer biology.

This review emphasizes the importance of understanding fetal programming of disease risk and the need for comprehensive studies elucidating the molecular mechanisms linking embryonic mammary gland development to breast cancer susceptibility. By integrating genomic, epigenomic, and environmental data, researchers can identify critical windows of susceptibility and develop targeted interventions for the prevention of breast cancer.

Rodent models provide an invaluable tool for investigating complex phenomena such as fetal mammary gland development and carcinogenesis. Their genetic similarities with humans, coupled with the ability to manipulate environmental factors in tightly controlled laboratory settings, offer unique advantages that are impractical or unethical to pursue in human studies. However, it is crucial to acknowledge the substantial architectural, hormonal, and stromal disparities between mouse and human mammary glands. By considering these interspecies differences, we can effectively model diseases and pinpoint suitable interventions within human biology, all while harnessing valuable insights learned from mouse models. Furthermore, advancements in culture systems, such as the adult stem cell-derived mammary mini gland culture, offer non-invasive and ethically sound platforms to study complex biological processes, including hormone responsiveness and oncogene-induced tumor initiation. Overall, the integration of rodent models and innovative culture systems presents promising avenues for unraveling the molecular mechanisms underlying the impact of maternal environments on offspring susceptibility to breast cancer in adulthood, ultimately contributing to the advancement of preventative and therapeutic strategies.

Overall, the reviewed literature underscores the necessity of a multifaceted approach that considers both genetic and environmental influences on breast cancer risk, with implications for public health initiatives, clinical practice, and future research direction.

## Data Availability

Not Applicable.

## Abbreviations

The following abbreviations are used in this manuscript:

APC	Adenomatous polyposis coli
BMP	Bone morphogenetic protein
DOHaD	Developmental origins of health and disease
ER	Estrogen receptor
FGF	Fibroblast growth factor
FGFR	Fibroblast growth factor receptor
HER2	Human epidermal growth factor receptor 2
Hh	Hedgehog
IDC	Invasive ductal carcinoma
ILC	Invasive lobular carcinoma
JAK	Janus kinase
*LEF1*	Lymphoid enhancer-binding factor 1
LRP	Low-density lipoprotein receptor protein
MDN	Methyl donor nutrients
NTD	Neural tube defects
*PTHrP*	Parathyroid hormone-related protein
PR	Progesterone receptor
STAT	Signal transducer and activator of transcription
TBX	T-Box transcription factor
TDLU	Terminal ductal lobular unit
TNBC	Triple-negative breast cancer
WNT	Wingless-type MMTV integration site family
